# Fracture Parameters and Cracking Propagation of Cold Recycled Mixture Considering Material Heterogeneity Based on Extended Finite Element Method

**DOI:** 10.3390/ma14081993

**Published:** 2021-04-16

**Authors:** Lei Gao, Xingkuan Deng, Ye Zhang, Xue Ji, Qiang Li

**Affiliations:** 1 Department of Civil and Airport Engineering, Nanjing University of Aeronautics and Astronautics, 29 Jiangjun Road, Nanjing 211106, China; glzjy@nuaa.edu.cn (L.G.); zhangye@nuaa.edu.cn (Y.Z.); jixue001@nuaa.edu.cn (X.J.); 2 College of Civil Engineering, Nanjing Forestry University, Nanjing 210037, China; liqiang2526@njfu.edu.cn

**Keywords:** cold recycling mixture, mixed-mode fracture performance, fracture parameters, cracking propagation, Arcan test

## Abstract

Cold recycled mixture (CRM) has been widely used around the world mainly because of its good ability to resist reflection cracking. In this study, mixed-mode cracking tests were carried out by the designed rotary test device to evaluate the cracking resistance of CRM. Through the finite element method, the heterogeneous model of CRM based on its meso-structure was established. The cracking process of CRM was simulated using the extended finite element method, and the influence of different notch lengths on its anti-cracking performance was studied. The results show that the mixed-mode fracture test method can effectively evaluate the cracking resistance of CRM by the proposed fracture parameters. The virtual tests under three of five kinds of mixed-cracking modes have good simulation to capture the cracking behavior of CRM. The effect of notch length on the initial crack angle and the crack propagation process of the CRM is mainly related to the distribution characteristics of its meso-structure. With the increase of the proportion of Mode II cracking, the crack development path gradually deviates, and the failure elements gradually increase. At any mixed-mode level, there is an obvious linear relationship between the peak load, fracture energy, and the notch length.

## 1. Introduction

Cold recycling is a kind of asphalt pavement recycling technology that has the technical characteristics of construction under normal temperature, so it has positive significance for resource conservation and environmental protection. As a result of its low cost and sustainable characteristics, this technology has been widely used around the world. Usually, reclaimed asphalt pavement (RAP), emulsion, water, additional aggregate, and additives are mixed to form rehabilitated pavement without the application of heat [1]. The repaired pavement can well resist the reflection cracking from underlying concrete, which is the key to the success of cold recycled mixture (CRM) [2]. Therefore, it is necessary to understand the cracking behavior and mechanism of CRM.

According to fracture mechanics, the cracking types of asphalt mixture can be divided into Mode I (opening), Mode II (sliding), and their combination. The combination of both is the mixed-mode cracking of asphalt mixture. There have been a lot of research studies on the test methods of asphalt mixture cracking [3,4,5,6,7]. Most of them are focused on Mode I cracking of asphalt mixture, and less attention is paid to Mode II cracking. Gao [8] had designed the Arcan testing device which can simulate five levels of mixed-mode cracking for asphalt concrete. Through a laboratory Arcan test combined with the digital image correlation (DIC) system, the mixed-mode cracking behavior of asphalt mixture can be effectively analyzed. However, due to the time-consuming and special devices needed in the laboratory Arcan test, the numerical simulation method is usually needed to study the mixed-cracking behavior of materials.

A three-dimensional numerical model of asphalt mixture was established by the discrete element method, and the fracture performance of CRM and a hot-mix asphalt mixture was compared [9]. The cohesive zone model (CZM) of cold in-place recycling mixture was established by the finite element method (FEM). The model has the potential capability to obtain the fracture process of Arcan specimen [10]. However, using the CZM model requires defining the cracking propagation path in advance, which doubtlessly has a great influence on the prediction of asphalt mixture crack trajectory. The extended finite element method (XFEM) has an obvious advantage in the study of fracture problems because it does not rely on mesh generation and does not need to define the propagation path of cracks [11]. XFEM has been used to study the crack resistance of a variety of materials [12,13,14]. In order to study the mechanism of reflective cracks on an asphalt concrete surface, the XFEM model was established, and the effects of temperature and traffic load were considered. The effects of initial cracking lengths and inclined degrees of initial crack on crack initiation and crack propagation were analyzed [15]. The stress distribution, crack initiation temperature, crack opening displacement, and crack propagation path of asphalt pavement with three different overlay thicknesses were studied [16]. Thus, the numerical simulations of mixed-mode cracking for mixtures could be better investigated by XFEM.

In this study, the overall objective is to analyze the mixed-mode cracking parameters and cracking propagation of CRM through the combination of Arcan test and FEM. Furthermore, the specific objectives of this paper are as follows:

(1) Obtain the fracture parameters such as peak load, fracture energy, and crack angle using the Arcan test method to study the cracking resistance of CRM;
(2) Verify the mixed-cracking simulation result of CRM with the fracture parameters from the laboratory Arcan test;
(3) Analyze the cracking process of CRM according to the stress distribution at different times in the virtual test;
(4) Investigate the anti-cracking mechanism of CRM with the effect of different notch length on mixed-cracking resistance of CRM studied by FEM.

## 2. Materials and Methods

### 2.1. Materials and Specimen Forming

In this research, CRM were all designed according to the cold in-place recycled specification in Jiangsu Province, China [17]. The mix design results of CRM are shown in Table 1. Portland cement (Wuxi Yangshijin Construction Material Co., Ltd., Wuxi, China) was added at 1.5% by weight of RAP materials. In addition, 3% of mineral filler (Lingshou Runling Mineral Products Trading Co., Ltd., Shijiazhuang, China) was added, and the optimum moisture content was 4.3%. The square specimens of CR-20 mixture were obtained by a rutting test. Then, the high-precision marble cutting machine was used to cut a square surface of the specimens to obtain the Arcan specimen with a smooth surface. The size of the Arcan specimens is 80 mm × 80 mm × 50 mm. Finally, a 40 mm notch was cut in the center of one side of the square specimen to complete the molding of the Arcan specimen. Furthermore, in order to achieve the application of DIC technology to obtain the displacement data that need to be recorded during the test, speckles are formed on the surface of the Arcan specimen by black and white spray paints.

### 2.2. Arcan Configuration

**Figure 1:**
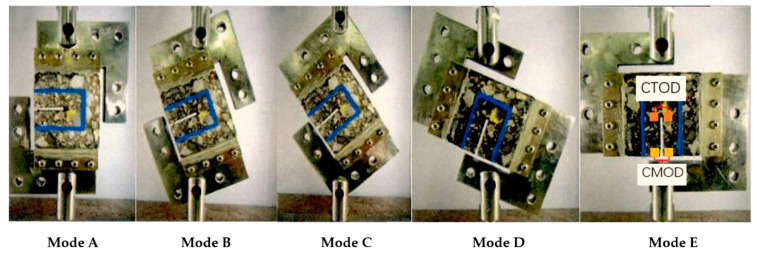
Arcan test configuration with five levels of mixed-mode cracking.

The Arcan testing device as shown in Figure 1, by adjusting the angle (90°, 67.5°, 45°, 22.5°, and 0°) between the loading direction and the initial notch, the stress modes of arcan specimens can be divided into Mode A (100% Mode I and 0% Mode II), Mode B (75% Mode I and 25% Mode II), Mode C (50% Mode I and 50% Mode II), Mode D (25% Mode I and 75% Mode II), and Mode E (0% Mode I and 100% Mode II), so as to simulate the stress state of asphalt mixture under different mixed-cracking modes. The Arcan test was carried out by a universal testing machine (Shanghai zhuozhilitian Technology Development Co., Ltd., Shanghai, China). The test temperature was set at −10 °C, and the tensile loading rate was set at 0.5 mm/min. The Arcan test has three key parameters: peak load, crack angle, and fracture energy. The peak load is the maximum load in the process of the Arcan test, which reflects the cracking strength of the asphalt mixture to a certain extent. The crack angle is the acute angle formed by the straight line connecting the crack starting point with the crack end point and the cutting direction after the specimen is damaged. Fracture energy is a comprehensive index to evaluate the anti-cracking performance of CRM. In the Arcan test, the measured displacement data mainly include load line displacement (LLD), crack mouth opening displacement (CMOD), and crack tip opening displacement (CTOD). The fracture energies corresponding to different displacement data are *G*_f-LLD_, *G*_f-CMOD_, and *G*_f-CTOD_. The specific calculation methods of three kinds of displacement fracture energy used in this paper are as follows:

(1) $$G_{f-LLD}=\frac{\int^{}Pdu_{1}}{A_{\mathrm{lig}}},$$

(2) $$G_{f-CMOD}=\frac{\int^{}Pdu_{2}}{A_{\mathrm{lig}}},$$

(3) $$G_{f-CTOD}=\frac{\int^{}Pdu_{3}}{A_{\mathrm{lig}}},$$

where *P* is the load; *A*_lig_ is the ligament area; *du*_1_, *du*_2_, and *du*_3_ are changes in *LLD*, *CMOD*, and *CTOD* respectively; *G*_f-LLD_ is the fracture energy from the *LLD* measurement; *G*_f-CMOD_ is the fracture energy from the *CMOD* measurement, and *G*_f-CTOD_ is the fracture energy from the *CTOD* measurement.

### 2.3. Numerical Simulation Method

#### 2.3.1. Homogenization Method of Composite Materials

**Figure 2:**
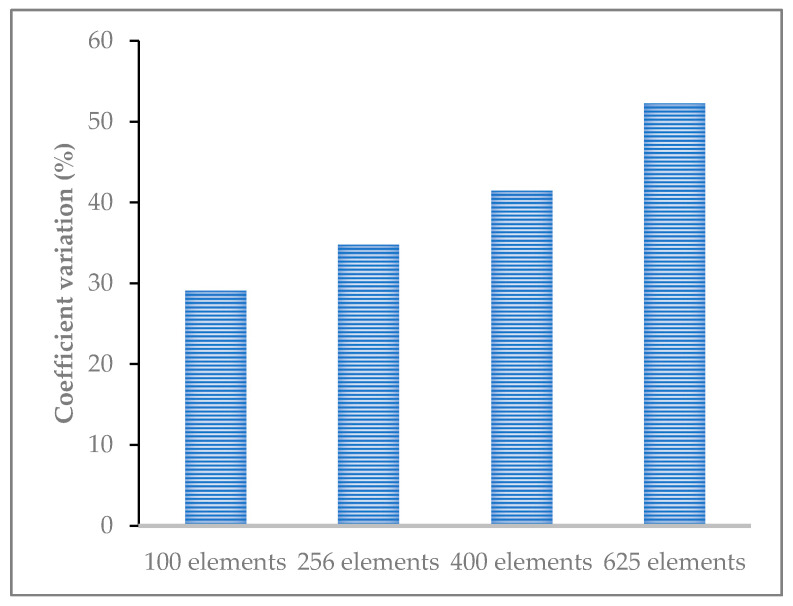
The results of coefficient variation calculated by different element size division.

Since the cracking of CRM in low temperature (especially subzero temperature) is mainly brittle, the elastic material characteristics are considered in the finite element simulation. There are two basic methods to homogenize the elastic parameters of composite materials: a series model (Voigt model) [18,19] and parallel model (Reuss model) [20,21]. The parallel model can ensure the deformation compatibility of each component in the material. In addition, the homogenization method of material parameters is needed to determine the element size. The selection of cell size was determined by moving window technology. The images were divided into 100, 256, 400, and 625 windows. The coefficient variation of the percentage of coarse aggregate in the window represents the uniformity of the material within the unit size. As can be seen from Figure 2, the smaller the unit size, the larger the coefficient variation is. Based on the representativeness of material sampling and the accuracy requirements of numerical simulation, this study selected 400 equal parts for element division.

The equivalent elastic modulus and equivalent shear modulus of each element in the parallel model are defined as follows:

(4) $$\{\begin{array}{c} E^{*}=\sum_{i=1}^{n}C_{i}E_{i}\\ G^{*}=\sum_{i=1}^{n}C_{i}G_{i} \end{array},$$

where E* is the equivalent elastic modulus; G* is the equivalent shear modulus; *C_i_* is the volume fraction of the component *i*; *E_i_* is the elastic modulus of the component *i*; and *G_i_* is the shear modulus of the component *i*.

According to the relationship between shear modulus and Poisson’s ratio, the equivalent Poisson’s ratio of the element can be deduced as follows:

(5) $$\mu^{*}=\frac{E^{*}}{2G^{*}}-1=\frac{\sum_{i=1}^{n}C_{i}E_{i}}{\sum_{i=1}^{n}C_{i}\frac{E_{i}}{1+\mu_{i}}}-1,$$

where μ* is the equivalent Poisson’s ratio, and *μ_i_* is the Poisson’s ratio of the component *i*.

#### 2.3.2. Model Building

**Figure 3:**
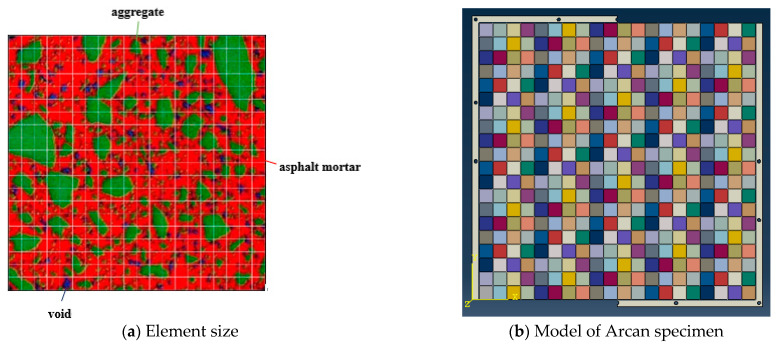
Schematic diagram of FEM model based on homogenization method.

According to the stress characteristics and the size of CRM specimen, the finite element model of the testing device was established. The establishment of the digital model of asphalt mixture, and its meso-structure should be considered [22]. Through the homogenization method of composite materials, the heterogeneous model of CRM based on its meso-structure was established. The continuous sectional image of CRM was obtained by CT scanning and digital image processing technology, and the image was divided into 400 elements, as shown in Figure 3a. The volume fraction of each component (emulsified asphalt mortar, aggregate, and voids) along the height direction of the specimen under the area of the element was calculated first, and then, the elastic parameters were determined by the homogenization method of composite materials. In ABAQUS 6.14-2 software, 400 two-dimensional CPE4R elements were established, each element was set as a separate section, and the calculated equivalent elastic parameters were input into the corresponding element. The established heterogeneous model is shown in Figure 3b. By simulating the initial notch with one-dimensional components without material characteristics, the Arcan digital specimens with different notch lengths were established. Through the XFEM method, the cracking process and mixed-mode cracking behavior of CRM under five different types of loads were obtained. According to the experimental results of Paulino, Kim, and Gao [7,23,24], the values of elastic parameters used in this paper are as follows: E = 42.0 GPa, μ = 0.15 for aggregates, and E = 0.185 GPa, μ = 0.25 for emulsified asphalt mortar. The fracture parameters for each element used in the XFEM method were obtained by the Arcan test under Mode A cracking mode, and their values are as follows: the tensile strength of 3.92 MPa and the fracture energy of 275 J/m^2^.

## 3. Results and Discussion

### 3.1. Mixed-Mode Cracking Test Results

**Figure 4:**
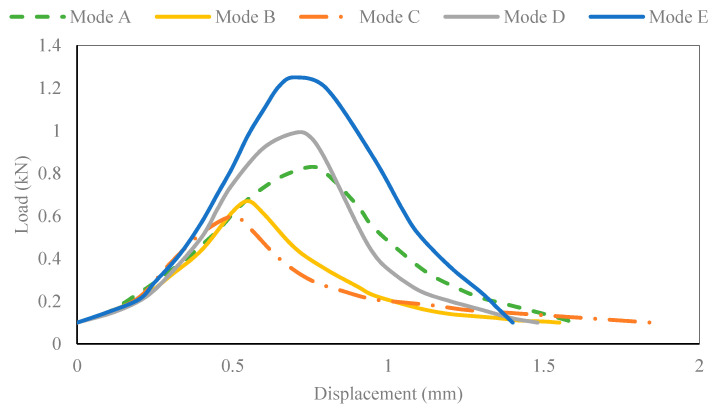
Load–displacement curves of CR-20 mixture in different mixed-mode levels.

The load displacement curve of the CR-20 mixture is shown in Figure 4. The ordinate axis corresponds to the tensile load, and the abscissa axis corresponds to the axial load displacement of the Arcan specimen. To some extent, the peak load can reflect the cracking resistance strength of CRM at a certain mixed-mode level. The fracture strength of the CR-20 mixture is affected by the cracking mode. The peak load of the CR-20 mixture reaches the minimum value at the mixed-mode level of Mode C, and it reaches the maximum value at the mixed-mode level of Mode E. Therefore, the crack resistance strength of the CR-20 mixture in the Arcan test is related to the stress mode. Under the mixed-mode level of Mode C, the peak load is the smallest and it cracks the most easily; at the mixed-mode level of Mode E, the peak load is the largest and the most difficult to crack. It can be seen that the fracture process of the CR-20 mixture under the five mixed-cracking modes shows certain softening characteristics. However, the softening characteristics of different loading modes are obviously different. The loading mode of Mode E (pure shear cracking mode) has the maximum peak load. The corresponding fracture curve decreases fastest after the peak load, and the characteristics of brittle fracture are the most obvious. The peak load of the Mode D fracture curve is the second, and the descending speed after the peak load is only next to Mode E. The fracture curve of Mode C has the minimum peak load, the decline rate is the slowest after the peak load, and the softening process is the most obvious in the fracture process. The peak load of the Mode B fracture curve is only greater than that of Mode A, and the decline speed of the fracture curve after the peak load is the second slowest. Therefore, the greater the crack resistance of CRM in a certain mixed-mode level, the greater the possibility of brittle fracture.

**Figure 5:**
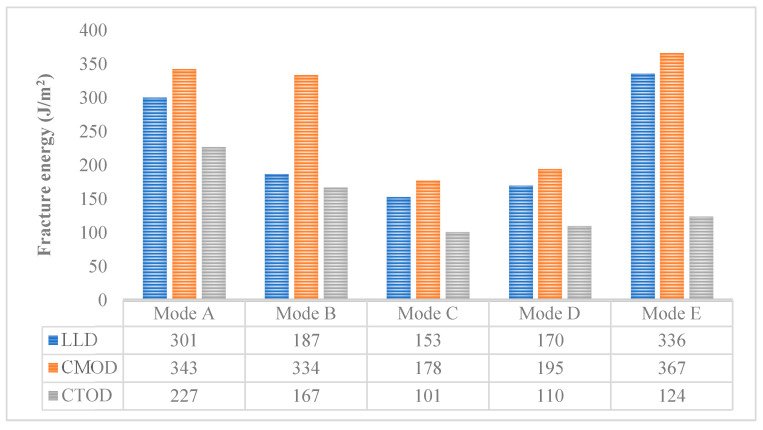
G_f-LLD_, G_f-CMOD_, and G_f-CTOD_ of CR-20 mixture.

Figure 5 summarizes the fracture energy of CR-20 mixture in five different mixed-mode levels. It can be seen that the three kinds of displacement fracture energies of the CR-20 mixture are sensitive to five kinds of mixed-mode cracking levels. In the cracking tests, the three kinds of displacement fracture energies of the CR-20 mixture have the same law: with the proportion of Mode I cracking reduced from 100% to 50%, the fracture energy decreased gradually; but with the proportion of Mode I cracking decreased from 50% to 0%, the fracture energy increased gradually. In addition, three different displacement fracture energies can be used to study the crack resistance of CRM. In any mixed-mode level, the order of displacement fracture energy is as follows: G_f-CMOD_ > G_f-LLD_ > G_f-CTOD_. Therefore, according to the results of G_f-CTOD_, the CR-20 mixture has the best crack resistance under Mode A, followed by Mode E, then Mode B and Mode D. Under Mode C, the crack resistance of the CR-20 mixture is the worst.

The corresponding fracture angles of the CR-20 mixture under different mixed-mode levels (Mode A, Mode B, Mode C, Mode D, and Mode E) are 6°, 15°, 29°, 45°, and 50° respectively. It can be seen that the crack angle of CRM in the Arcan test has a clear relationship with the mixed-cracking level. With the increase of the ratio of Mode II cracking, the crack angle of the CR-20 mixture will increase correspondingly. Therefore, according to the corresponding relationship between the crack angle and the mixed-cracking level in the Arcan test, the types of reflection cracking (tensile, shear, and mixed-mode) of the actual cold recycling pavement can be determined, so as to judge the failure stress mode of the actual pavement.

### 3.2. Verification of Numerical Simulation Results

**Figure 6:**
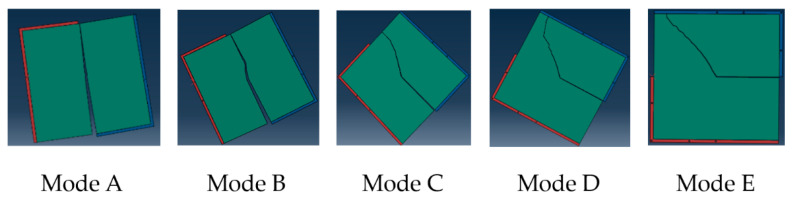
Virtual cracking results of the CR-20 mixture under five different mixed-mode levels.

Figure 6 shows the cracking results of the virtual specimen of the CR-20 mixture with a 40 mm notch under five different mixed-mode levels. The crack angle is the acute angle formed by the straight line connecting the crack starting point with the crack end point and the cutting direction after the specimen is damaged. According to the simulation results, the corresponding crack angles of the CR-20 mixture under five different mixed-mode levels (Mode A, Mode B, Mode C, Mode D, and Mode E) are 3°, 11°, 23°, 38°, and 44° respectively. Compared with the laboratory test results, it can be concluded that the virtual Arcan test has reasonable results.

**Figure 7:**
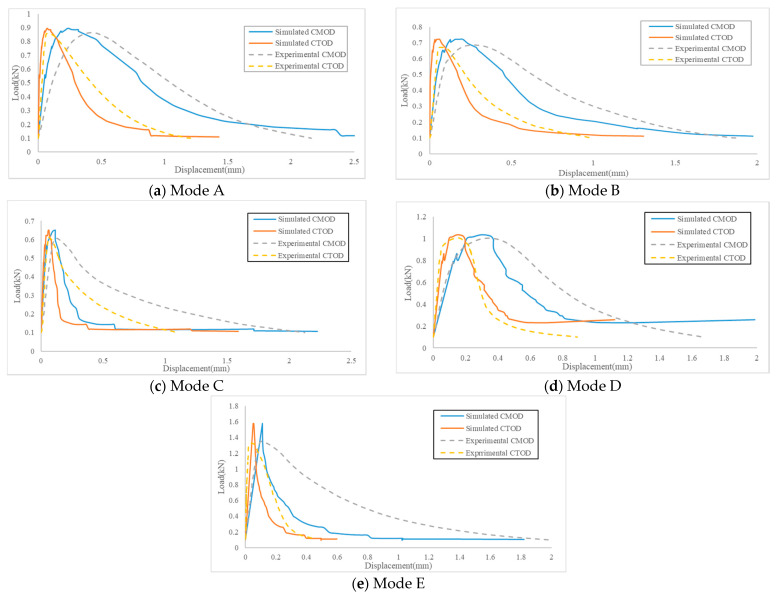
Comparison of simulated results and experimental results of the CR-20 mixture with 40 mm notch under five kinds of mixed-mode levels.

The LLD of the Arcan test cannot directly reflect the deformation of the specimen, but CMOD and CTOD can directly and accurately determine the deformation of the Arcan specimen and the specific development stage of the Arcan specimen. Therefore, using the P-CMOD curve and P-CTOD curve can better verify the accuracy of simulated results. Figure 7a–e shows the load–displacement curve comparing the simulated results with the experimental results. Figure 7a corresponds to the Mode A cracking level, in which the simulated results of the P-CTOD curve are closer to the experimental curve, and the peak load of the Arcan test and the development trend of CTOD in the test process are close to the experimental results. The results of the P-CMOD curve for the peak load are consistent, but the calculation results of CMOD in the test process are different from the experimental results. Figure 7b corresponds to the Mode B cracking level; similar to Figure 7a, the simulated result of the P-CTOD curve is better than that of P-CMOD. The main reason for this phenomenon is that CTOD is only affected by the deformation at the notch tip of the Arcan digital specimen, and the factors considered are single. However, CMOD is affected by the deformation of the notch tip and the development of cracks, so the situation of CMOD is more complex, which leads to the deviation of CMOD results of the Arcan virtual test larger than the tip displacement. Figure 7c corresponds to the Mode C cracking level; the peak load result is slightly larger than the laboratory test result, and while the corresponding CMOD and CTOD are close to the experimental result, the overall CMOD and CTOD results are quite different from the experimental curve. Figure 7d corresponds to the Mode D cracking level, which is similar to Figure 7a,b. The similarity of the peak load results is the best, and the similarity between the simulated result of the P-CTOD curve and experimental result is the highest. Figure 7e corresponds to the Mode E cracking level; similar to Figure 7c, its peak load is greater than the experimental results, and the values of CMOD and CTOD are quite different from the laboratory values, so it can not be used as a substitute for a laboratory test.

The simulated fracture energy of the Arcan specimen with a 40 mm notch under five different mixed-mode levels was compared with the laboratory results, and the reliability of the simulation results of the virtual test was analyzed. According to the definition and calculation method of the Arcan test fracture energy, the results of G_f-CMOD_ and G_f-CTOD_ are shown in Table 2. The results show that the virtual test under Mode A, Mode B, and Mode D has good reliability, and the relative error between simulation results and laboratory results is less than 10%. Among them, the accuracy of the virtual test results is the best under the Mode E cracking level. The simulation results of Mode C and Mode E are quite different from the laboratory results. Therefore, the virtual Arcan test results under the Mode C and Mode E cracking levels cannot be used for analysis.

### 3.3. Analysis of Numerical Test Results

**Figure 8:**
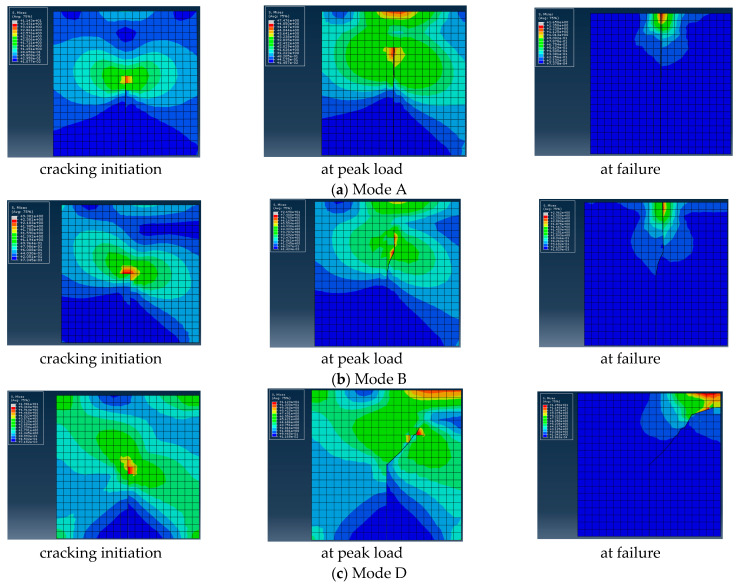
The stress distributions at different stages of loading for three levels of mixed-mode cracking.

In order to analyze the cracking process of the CR-20 mixture, the stress distributions at the cracking initiation, peak load, and failure moment of digital specimen were derived from ABAQUS, as shown in Figure 8. The difference of stress distribution under the three kinds of cracking modes is mainly concentrated on the stress value and crack tip position. In the cracking process of three kinds of cracking modes, stress concentration exists at the crack tip. The partial discontinuity stress gradient was due to the large difference of elastic parameters between some adjacent elements. The peak loads of the three kinds of cracking modes (Mode A, Mode B, and Mode D) were 885, 721, and 1035 N. The load at the initial cracking moment was 754, 586, and 894 N, accounting for 85.2%, 81.3%, and 86.4% of the peak load, respectively. The load at the initial cracking of Mode B accounts for the smallest proportion of the peak load, and the load at the initial cracking of Mode D accounts for the largest proportion of the peak load. Therefore, the Mode B cracking mode is more prone to initial cracking.

**Figure 9:**
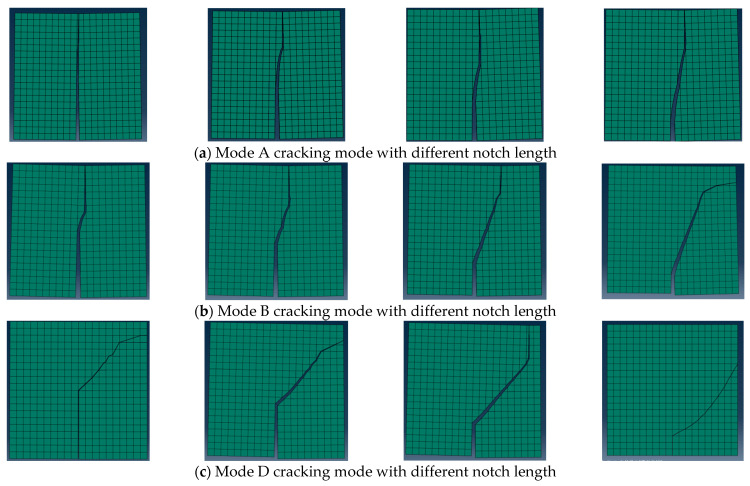
Numerical Arcan test results of mixed-mode cracking with different notch lengths (40, 30, 20 and 10 mm).

In order to further analyze the anti-cracking performance of the CR-20 mixture under three kinds of cracking modes (Mode A, Mode B and Mode D), a numerical Arcan test was carried out on the digital specimens of CRM with different notch lengths (10, 20, 30 and 40 mm). The numerical results of mixed-mode cracking test with different notch lengths are shown in Figure 9. For the Mode A cracking mode, the corresponding crack angles of the virtual test with different notch lengths (40, 30, 20 and 10 mm) are 2.9°, 4.6°, 3.8° and 6.5° respectively. For the Mode B cracking mode, the corresponding crack angles corresponding to different notch lengths are 5.7°, 9.1°, 18.4° and 33.7° respectively. For the Mode D cracking mode, the crack angles corresponding to different notch lengths are 51.3°, 45.0°, 29.2° and 41.6° respectively. For any initial notch length, the order of crack angle is as follows: Mode A < Mode B < Mode D. It is difficult to describe the relationship between the crack angle and the notch length directly through the crack angle calculated from the starting point and the crack end point, which is because the crack propagation path is also related to the distribution characteristics of its meso-structure. In order to further analyze the relationship between the crack angle and notch length, the initial angle of the mixed-mode crack is calculated. Corresponding to the different initial notch lengths (40, 30, 20, 10 mm), the crack initiation angles in Mode A are 2.8°, 8.3°, 10.0° and 10.3°, the crack initiation angles in Mode B are 12.6°, 15.4°, 17.3°, and 18.4°, and the crack initiation angles in Mode D are 40.7°, 41.2°, 45.3° and 59.5°, respectively. It can be found that for the three mixed-cracking modes, the initial angle of mixed-mode crack increases gradually with the decrease of the notch length.

**Figure 10:**
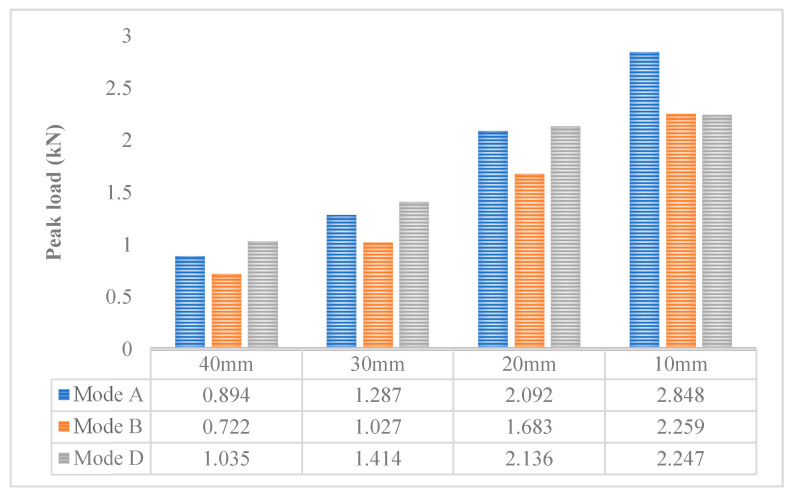
The peak load results of the virtual Arcan test with different notch lengths and different mixed-mode levels.

Figure 10 shows the peak load results of virtual Arcan tests with different mixed-mode levels and different notch lengths. It can be seen that for the three different mixed-mode levels, the smaller the notch length, the greater the peak load result is. This phenomenon shows that for the three different cracking modes, the larger the notch length, the easier it is for the specimen to crack. For the virtual Arcan test of 20 mm, 30 mm, and 40 mm notch length, the order of peak load of different mixed-mode levels is Mode D > Mode A > Mode B. This is in accordance with the results of the laboratory tests. The order of peak load under the 10 mm notch length is Mode A > Mode B > Mode D. The peak load value of the Mode D cracking mode is the smallest among the three cracking modes under the 10 mm notch length. This is because under the Mode D cracking mode, the crack propagation path of the CRM specimen with the 10 mm notch length is the shortest. The results show that the peak load of the Mode A cracking mode and the 10 mm notch length is the largest, which verifies the previous conjecture. It shows that the peak load results of the test are related to the crack development path of the specimen.

**Figure 11:**
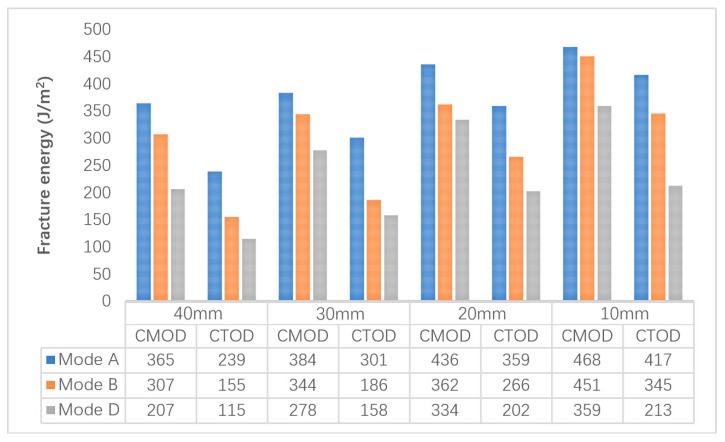
The results of G_f-CMOD_ and G_f-CTOD_ of a virtual Arcan test with different notch length and different mixed-cracking modes.

Fracture energy is a reliable and comprehensive parameter to evaluate the crack resistance of materials [22]. G_f-CMOD_ and G_f-CTOD_ were used to further evaluate the crack resistance of the CR-20 mixture. The G_f-CMOD_ and G_f-CTOD_ results of the virtual Arcan test under different notch lengths and different mixed-mode levels are shown in Figure 11. It can be found that the values of G_f-CMOD_ and G_f-CTOD_ increase with the decrease of notch length at any mixed-mode level. The smaller the initial notch length, the greater the fracture energy required for the failure of the CR-20 mixture under the determined mixed-cracking mode. Under the selected initial notch length, the order of G_f-CMOD_ and G_f-CTOD_ values is Mode A > Mode B > Mode D, which is consistent with the experimental results. It also reflects that the fracture energy of the Arcan test is a more comprehensive evaluation index, and it is not affected by the length of the notch to a certain extent. Among all the fracture energy results, the fracture energy required for the Arcan specimen with an initial notch length of 10 mm is the largest under the Mode A cracking mode. This is because the Arcan specimen with an initial notch length of 10 mm has the longest crack development path under the Mode A cracking mode, which also confirms the previous peak load results of the virtual Arcan test. The fracture energy of the specimen with an initial notch length of 40 mm is the minimum at the Mode D cracking level. The fracture energy of the specimen with an initial notch length of 40 mm is the minimum at the Mode D cracking level, which is also related to the crack propagation path. It can be concluded that the CR-20 mixture with a 40 mm notch length under the Mode D cracking mode is most likely to crack. Meanwhile, under the Mode A cracking mode, the CR-20 mixture with a 10 mm notch length is the most difficult to crack.

## 4. Conclusions

In this paper, the cracking resistance and cracking behavior of a CR-20 mixture under different mixed-cracking modes were studied by the Arcan test method and FEM. The mixed-mode cracking test of a CR-20 mixture was carried out with an Arcan testing device, and the simulation of the Arcan test of the CR-20 mixture was carried out by FEM. The reliability of the simulation was verified by comparing the simulation results with the experimental results. Based on the results and analysis, the following conclusions can be made:

1. The fracture parameters such as peak load, crack angle, and fracture energy were obtained by Arcan tests. The order of displacement fracture energy is G_f-CMOD_ > G_f-LLD_ > G_f-CTOD_. According to the result of G_f-CTOD_, the order of crack resistance of the CR-20 mixture under five kinds of mixed-cracking modes is as follows: Mode A > Mode E > Mode B > Mode D > Mode C.
2. The virtual Arcan configuration and two-dimensional digital specimen were set up in the ABAQUS software by using FEM. Considering the material heterogeneity and combined with the XFEM cracking mechanism, the cracking behavior of the CR-20 mixture under different mixed-mode levels was studied.
3. According to the reliability of the virtual test, Mode A, Mode B, and Mode D cracking has better simulation in five kinds of mixed-mode virtual cracking tests. Furthermore, the relative error of the Mode D cracking mode is the smallest among the three kinds of mixed-cracking modes.
4. With the increase of the proportion of Mode II cracking, the development path of cracks gradually deviates, and the number of failure elements increases. In any mode of cracking process, the stress concentration exists at the crack tip. The cracking propagation of the CR-20 mixture is not only affected by the length of the initial notch but also related to the distribution characteristics of its meso-structure.
5. There is a certain relationship between the initial notch length and the initial crack angle, peak load, and fracture energy. Through two kinds of fracture energy, the anti-cracking performance of the CR-20 mixture with different notch lengths under different mixed-cracking modes was evaluated. The results show that the CR-20 mixture with a 10 mm initial notch length has better cracking resistance under the pure tensile stress mode.

## Figures and Tables

**Table 1:** Mix design results of the CR-20 mixture.

Mixtures	CR-20	
Optimal Asphalt Content (%)	3.5(CSS-1)	
Sieve Size (mm)	Passing Percent (%)	Limits
26.5	100	100
19	96.7	90–100
16	92.4	-
13.2	84.3	-
9.5	70.1	60–80
4.75	50	35–65
2.36	36	20–50
1.18	22.2	-
0.6	14.5	-
0.3	8.3	3–21
0.15	6.1	-
0.075	3.7	2–8

**Table 2:** Comparison between experimental fracture energies and the simulated results.

Fracture Energy		Mixed-Mode Level				
		Mode A	Mode B	Mode C	Mode D	Mode E
G_f-CMOD_ (J/m^2^)	Simulated G_f-CMOD_	365	307	127	207	215
	Experimental G_f-CMOD_	343	334	178	195	367
	Relative error	6.41%	8.08%	28.65%	6.15%	41.42%
G_f-CTOD_ (J/m^2^)	Simulated G_f-CTOD_	241	155	73	116	67
	Experimental G_f-CTOD_	227	167	101	110	124
	Relative error	6.17%	7.18%	27.72%	5.45%	45.97%

## Data Availability

Data available on request due to restrictions eg privacy or ethical. The data presented in this study are available on request from the corresponding author. The data are not publicly available due to these data are part of ongoing research.
